# Extended-Spectrum Beta-Lactamase-Producing and Carbapenem-Resistant *Enterobacterales* in Companion and Animal-Assisted Interventions Dogs

**DOI:** 10.3390/ijerph182412952

**Published:** 2021-12-08

**Authors:** Emanuela Roscetto, Chiara Varriale, Umberto Galdiero, Camilla Esposito, Maria Rosaria Catania

**Affiliations:** Department of Molecular Medicine and Medical Biotechnology, University of Naples Federico II, 80131 Naples, Italy; emanuela.roscetto@unina.it (E.R.); chiaravarriale98@gmail.com (C.V.); galdiero.umberto@libero.it (U.G.); cam.esposito.ce@gmail.com (C.E.)

**Keywords:** animal-assisted interventions, companion dog, extended-spectrum beta-lactamase, carbapenem-resistant *Enterobacterales*, One Health

## Abstract

Animal-assisted interventions (AAIs) are being implemented in many countries for the beneficial effects they have on humans. Patients involved in AAI are often individuals at greater risk of acquiring infections, and these activities involve close contact between humans and animals, as is the case with humans living with a pet. The spread of multidrug-resistant *Enterobacterales* is a serious problem for human health; an integrated One Health strategy is imperative to combat this threat. Companion dogs can be a reservoir of multidrug-resistant pathogens, and animal-to-human transmission could occur during AAI sessions. The aim of this review was to collect the available data on the carriage of extended-spectrum beta-lactamase-producing and carbapenem-resistant *Enterobacterales* in companion dogs and in an AAI context. Several papers have generally addressed the issue of microbial transmission during AAIs. Studies on the intestinal carriage of extended-spectrum beta-lactamase and/or carbapenem-resistant *Enterobacterales* have mainly been conducted in companion animals while few data are available on the carriage in dogs participating in AAI sessions. This review aims to draw attention to the antibiotic resistance problem in a One Health context and to the importance of extending infection control measures to this human–animal interface, to keep the balance of benefits/risks for AAIs shifted towards the benefits of these activities.

## 1. Introduction

The One Health strategy is based on the awareness that human, animal, and environmental health are closely connected, so that global health and the sustainability of life on our planet can only be achieved through integrated multidisciplinary interventions [1]. Among the main areas of intervention in which this approach is indispensable, there is the fight against the spread of antibiotic resistance and the control of zoonoses. Microorganisms are, in fact, a link between animals, humans, and the environment.

The inappropriate or excessive use of antibiotics in human and veterinary medicine and in food production has favored the spread of bacteria that are resistant to multiple classes of drugs at the same time [2]. Due to the availability of a limited number of new drugs, all belonging to old classes of antibiotics, humans risk losing the battle against diseases of bacterial etiology. In the absence of an effective One Health approach [3], the World Health Organization (WHO) has predicted a post-antibiotic era: in 2050, drug-resistant pathogens could cause 10 million deaths each year from untreatable bacterial infections [4]. In 2017, the WHO published a list of pathogens for which there is an absolute need to develop new drugs [5]. Extended-spectrum beta-lactamase (ESBL)-producing and carbapenem-resistant *Enterobacterales* fall into the most critical group of multi-resistant pathogens.

The risk of transmission of antibiotic-resistant pathogens from food to humans has been extensively investigated [6,7], while non-foodborne pathways of transmission have received less attention. An alternative way of resistant bacteria acquisition might be direct interaction with animals, such as pets and animals participating in animal-assisted interventions.

Animal-assisted interventions (AAIs) include a range of activities based on the interaction of human-to-companion animal. Animal-assisted therapy (AAT), animal-assisted education (AAE), animal-assisted activity (AAA) and, more recently, animal-assisted coaching (AAC) are forms of animal-assisted interventions.

AAT is a targeted, planned, and structured intervention in which an animal is integrated into a treatment process [8]. AAT is guided by formally trained health, education, and human services professionals and its goals are represented by the improvement of the physical, cognitive, behavioral, and/or socio-emotional functioning of the human recipient [9]. The intervention progress of AAT has to be evaluated and included in professional documentation.

AAE is an oriented, planned, and structured intervention guided by a general education or a special education practitioner and focused on academic goals, prosocial skills, and cognitive functioning [9]. Student progress has to be both measured and documented.

AAA is an oriented and planned informal intervention conducted by the human–animal team for motivational, educational, and recreational purposes to improve the quality of life [9]. There are no treatment goals for this form of intervention. AAAs are generally conducted by specially trained professionals, paraprofessionals, or volunteers [8].

AAC is a targeted, planned, and structured intervention guided by formally trained professional coaches. The goals are represented by the enhancement of recipient’s personal growth, social skills, and/or socio-emotional functioning of the coach or client [9]. The outcomes must be measured and included in the professional documentation.

**Figure 1:**
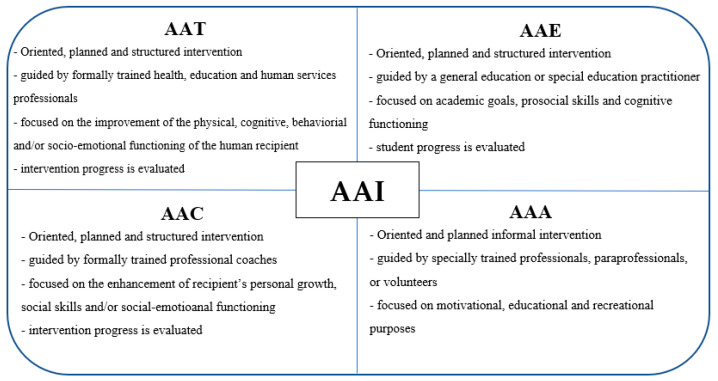
Key features of the various forms of Animal-assisted intervention (AAI).

The main characteristics of the various types of AAIs are summarized in Figure 1.

The beneficial effect of AAI on the physical and psychological conditions of the human recipient has been reported for various pathologies and conditions, especially for chronic and progressive neurological diseases [10,11] and psychiatric disorders [12]. Examples of AAI are represented by occupational therapy for young people with autism [13], physical therapy in geriatric populations [14], speech therapy for patients with acquired communication disorders [15], human education programs [16], and reading programs [17]. Furthermore, crisis response visits, activities with hospital patients and nursing home residents [9], and delinquent youth visits [18] are included in AAIs.

Animals commonly involved in AAI are dogs, cats, rabbits, guinea pigs, horses, birds, cows, and other farm animals [19]. The animal can participate in AAI through various tasks depending on the aim of the intervention [20]. Dogs are the most frequently used animals.

The benefits deriving from these interventions are so evident that the number of AAIs is constantly increasing. AAIs are usually carried out in healthcare facilities and mainly involve patients belonging to weaker categories (unhealthy persons, children, the elderly, people with disabilities). In this context, the close interactions between humans and animals can facilitate the transmission of potentially pathogenic microorganisms from animals to patients with risk factors for infections or vice versa. Enoch et al. (2005) described a case of a pet-therapy dog that acquired methicillin-resistant *Staphylococcus aureus* (MRSA) after visiting care-of-the-elderly wards [21]. In a study of 26 therapy dogs and 26 trainers, one dog was positive for *C. difficile* after the visit and one handler acquired MRSA [22]. However, these studies only suggest the potential risk of human–animal transmission. Several authors have assessed the carriage of zoonotic pathogens by therapy animals as an indirect evaluation of the risk of zoonotic transmission [23,24,25]. The purpose of this minireview is to evaluate the occurrence of ESBL-producing and carbapenem-resistant *Enterobacterales* in domestic and hospital-admitted dogs, and the spread of these resistant bacteria between dogs and humans.

## 2. Extended-Spectrum Beta-Lactamase-Producing and Carbapenem-Resistant *Enterobacterales* in Human Infections

*Enterobacterales*, such as *Escherichia coli* and *Klebsiella pneumoniae*, are part of the human gut microbiota; they are among the most frequent causes of infections associated with healthcare facilities [26] and are also responsible for many community-acquired infections (e.g., intestinal and urinary infections and sepsis). *Enterobacterales* are naturally sensitive to many classes of antibiotics, but the progressive acquisition of resistance determinants has led to the emergence of strains with a multidrug-resistance (MDR) phenotype. In fact, in recent decades, there has been a rapid increase in resistance to penicillins and cephalosporins due to the global spread of ESBLs; first in *K. pneumoniae,* then in other *Klebsiella* species, and finally in *E. coli* [27]. ESBLs are capable of degrading penicillins, cephalosporins (including third and fourth generation ones and those with anti-methicillin resistant *Staphylococcus aureus* activity) and monobactams, but not carbapenems. ESBL-producing *Enterobacterales* are widespread in the hospital setting but also in outpatient medicine [28]; in particular, *E. coli*-producing beta-lactamase CTX-M is observed with increasing frequency as a cause of urinary infections acquired in healthcare and outpatient settings. As in a vicious circle, the global spread of ESBLs has caused an increase in the use of carbapenems [29], which in turn has increased the selective pressure and thus facilitated the spread of carbapenem-resistant *Enterobacterales* (CRE). Resistance to carbapenems can be mediated by the production of β-lactamases with efficient carbapenemase activity (carbapenemase-producing CREs) or by the hyperproduction of β-lactamases with limited affinity and/or hydrolytic activity toward carbapenems, combined with porin mutations or overexpression of efflux pumps (non-carbapenemase-producing CRE). Currently, ESBLs and carbapenemases are the most clinically important enzymes for epidemiology and resistance implications. From an epidemiological point of view, these enzymes are of great importance since the corresponding genes are carried by transferable plasmids that can spread rapidly among enterobacteria [30]. In the Ambler classification system, carbapenemases are distributed in classes A, B, and D. Classes A and D enzymes are serine-carbapenemases, while class B includes metallo-beta-lactamases (MBLs). *K. pneumoniae* carbapenemases (KPCs) are the most common serine-enzymes among *Enterobacterales*. The most frequently identified MBLs are New Delhi metallo-beta lactamases (NMD), Verona integron-encoded metallo-beta-lactamases (VIM), and imipenemase metallo-β-lactamase (IMP), while in class D, carbapenemases of common detection are Oxacillinase (OXA)-48 and OXA-23 [31]. CREs, especially *K. pneumoniae*, have an elevated ability to cause outbreaks in health care settings; this has been reported in many EU countries [32,33,34,35,36,37,38,39]. The risk factors for the acquisition of CRE in a healthcare setting are similar to those for other multi-resistant bacteria and include hospitalization in the ICU, a prolonged stay in the ICU, a critical clinical situation, the presence of invasive medical devices, and previous antibiotic therapy [40,41]. Identification of CRE-colonized patients at admission is currently considered a standard-of-care for preventing and controlling the spread of these infections within care facilities and is recommended by guidelines in conjunction with isolation interventions aimed at preventing the cross-transmission of MDR strains [42]. On the other hand, it is known that colonization with MDR enterobacteria represents a risk factor for subsequent infection [43]. Colonization, mainly of the intestinal tract, with CRE is associated with high rates of infection (16.5% overall ranging from 0% to 89% in high-risk patients), particularly pneumonia, followed by urinary tract, bloodstream, surgical site, skin, and soft-tissue infections [43]. The eradication of CRE from the intestinal flora is very difficult. Rates of spontaneous decolonization vary in the different studies carried out [44,45] and cases of intestinal carriage of these bacteria have also been reported for over two years [45]. While carbapenem-resistant *K. pneumoniae* are frequently associated with the onset of outbreak in healthcare settings, carbapenem-resistant *E. coli* have a higher risk of spread at the community level [46]. Community-acquired CRE infections from patients who had had no relationship with care facilities in the previous three months have recently been reported in the EU [47]. There is growing evidence that extra-intestinal pathogenic *E. coli* or their resistance genes can be transmitted through the food chain from an animal food source, becoming part of the gut flora of healthy people who have not previously been exposed to care or treatment with antibiotics [48,49].

## 3. ESBL-Producing and Carbapenem-Resistant *Enterobacterales* in Companion Animals

Companion dogs are in close contact with humans, so it can be postulated that they may play a role in the transmission of MDR bacteria [50,51]. In fact, companion dogs mostly live in the same dwellings as their owners, who often caress and kiss them, and allow them to sleep in their beds and to lick them. In many cases, dogs can also come into contact with their owners’ food. 

Belas et al. (2001) [52] showed that simultaneous carriage of beta-lactam resistance genes between animals and their owners and possible fecal contamination of the environment increase interspecies transmission.

As part of a national surveillance program at 36 veterinary hospitals in South Korea, 315 companion dogs and 81 humans were screened for the presence of extended-spectrum cephalosporins (ESC)-resistant *Enterobacterales* [51]; the rates of ESC-resistant and ESBL-producing *E. coli* were 43.5% and 29.2%, respectively. Four *E. coli* isolates also produced the New Delhi metallo-beta-lactamase 5 (NDM-5) in addition to CTX-M-15. The rates of ESC-resistant and ESBL-producing *K. pneumoniae* were 8.3% and 7.6%, respectively. Of note is that five AmpC-producing *E. coli* isolates from two humans and three companion dogs were epidemiologically related, suggesting the possibility of human–animal transmission. Another Korean study reported a lower prevalence of ESBL-producing *E. coli* in companion dogs (18.9%) [53].

In order to assess the risk of human exposure to ESBL-producing *Enterobacterales*, it is important to consider not only the intestinal carriage of resistant bacteria by companion dogs, but also the continuity of such colonization. Baede et al. (2015) [54] showed a high prevalence of ESBL-producing *Enterobacterales* among healthy household dogs, but they were present in the dog’s gut for only a short period of time. Silva et al. (2018) [55] reported pneumonia due to *K. pneumoniae* producing extended-spectrum beta-lactamase CTX-M-15 in a 5-year-old domestic dog; after treatment with meropenem, the clinical conditions improved significantly, but the dog had persistent colonization by CTX-M-15 *K. pneumoniae* in the nasal and the rectal districts. The authors argued that prolonged carriage of MDR bacteria by companion animals may represent a risk condition for transmission to humans. Enteric carriage of ESBL-producing *E. coli* in companion dogs was also described in southeast Brazil, Germany, and Greece [56,57,58].

However, owners and other family members may themselves promote persistent pet colonization, either directly or indirectly through the household environment [59]. In support of this, in a pilot study, Ljungquist et al. (2016) [60] reported that intestinal transport of ESC-resistant *Enterobacterales* in dogs was more prevalent in families where a member was known to carry an ESC-resistant strain, than in families where there were no human carriers, indicating the bacterial transmission from human to dog. Similarly, Toombs-Ruane et al. (2020) [61] showed that, in some families, dogs carried the same ESBL-producing *E. coli* strain as the family member with a urinary tract infection. In another report, the co-carriage of ESC-*Enterobacterales* in owner–pet pairs was not found [62].

Another risk factor to consider is environmental exposure. Formenti et al. (2021) [63] demonstrated that the prevalence of ESC-resistant *E. coli* was significantly higher in domestic dogs that frequented extra-urban areas than in urban dogs [63].

The spread of CRE in humans poses a serious threat to public health. These threatening bacteria are beginning to be reported in companion animals, and all major classes of transmissible carbapenemase genes (KPC, NDM, VIM, IMP, OXA-L48, and OXA-23) have been described in companion dogs. Bandyopadhyay et al. (2021) detected genetically heterogeneous *E. coli*-producing metallo-beta-lactamases (MBLs) in companion dogs in India and recommend active surveillance measures to identify CRE transmission in pet animals [64]. NDM-5–producing *E. coli* have been reported in dogs in United States, United Kingdom, Italy, Algeria, South Korea, and Finland [65,66,67,68,69,70,71]. In the latter study, NDM-5-producing *E. coli* were identified from two dogs in one family; the family members were also screened for carbapenem-resistant bacteria and one of them was found to carry a strain related to those from the two dogs.

In a surveillance study on carbapenem-resistant Gram-negative bacteria in companion dogs, Cui et al. (2008) [72] reported the isolation of an *E. coli* strain producing NDM-1 and exhibiting an MDR phenotype to beta-lactams, quinolones, gentamicin, tetracycline, and phosphomycin. The identification of NDM-1 in MDR strains of *E. coli* from companion animals was also reported by Shaheen et al. (2013) [73].

OXA-48 beta-lactamase genes were identified in carbapenem-resistant *E. coli* from companion dogs in Algeria, France, and the United States and in *K. pneumoniae* and *Enterobacter cloaceae* from pets in Germany [57,74,75,76]; *E. coli* harboring the oxa-43 gene and a VIM-1-producing *K. pneumoniae* strain were isolated in pet dogs in Spain [77].

The risk of intestinal colonization in companion dogs increases when the dog is admitted to a veterinary clinic. Dogs can acquire CRE within a short time of hospitalization [78]. Although the colonization of animals in the study was generally short lived (almost all animals with intestinal carriage were negative within 4 months after hospital discharge), it poses a risk after dogs return to their homes.

## 4. ESBL-Producing and Carbapenem-Resistant *Enterobacterales* in Animal-Assisted Interventions Dogs

AAIs present various risk factors for the transmission of multi-drug-resistant bacteria. The benefits deriving from animal-assisted interventions are strictly dependent on the close interaction between patient and animal. The dogs participating in AAI are caressed, kissed, and placed on patient beds and the patient is licked by the animal [36]. Therefore, pets could act as mechanical vectors of canine and hospital pathogens and contribute to the transmission of these pathogens between patients or, in any case, within the hospital environment.

In 2006, in a study of 102 visitation dogs, ESC-*E. coli* were identified in three animals [79].

In a study of 98 dogs involved in AAIs, dogs were screened for various bacteria including ESBL and AmpC producer *E. coli* [80]. Colonization with *E. coli*-producing Amp-C appeared not to be related to exposure to health facilities, but to pre-visit antibiotic treatments and diet. ESBL-producing *E. coli* were not isolated. The correlation between colonization of pet therapy dogs with ESC-resistant *E. coli* and diet was also confirmed in another study in 2008 [81].

The transmission of potentially pathogenic bacteria between children and dogs during animal-assisted therapy was reported, but carriage was shown to be transient [82]. A dog participating in pet therapy was constantly colonized with *E. coli* but no dog-associated infections were reported. The authors suggested even washing the ward floors before the visit to prevent dogs from acquiring bacteria from the environment and passing them to patients. The importance of environmental exposure was highlighted by Lefebre et al. (2009) who showed that dogs participating in animal-assisted activities in hospitals became contaminated more frequently than those who visited other facilities [83].

In a study conducted in a Brazilian hospital that began dog-assisted therapy in 2014, dogs were screened before and after surgery for ESBLs, KPC, and NDM: no dogs were found with intestinal carriage of MDR-bacteria [84]. The nasal microbiota of patients and dogs during AAI was analyzed to verify the microbial sharing between animals and humans [85]. The composition of the nasal microbiota of dogs and pediatric patients was altered after AAI sessions, and the alteration was related to the duration and level of contact. This association between microbial sharing and level of interaction changed if the dog was decolonized before the visit. The study did not evaluate the presence of antibiotic resistance determinants.

An estimate of the eventual transmission of pathogens from dogs to patients during AAIs can be obtained by evaluating the infection rate at the hospital-level before and after pet therapy. In a pilot study carried out in Italy, Caprilli et al. (2006) [86] found that hospital infection rate did not increase and no new infections were recorded during AAIs. Similarly, Hardin et al. reported no documented infections whilst averaging 20,000 AAIs per year, where infection prevention and control programs were implemented and careful preparation of dogs, patients, operators, and the environment was carried out [87].

## 5. Conclusions

The spread of ESBL-producing and carbapenem-resistant Enterobacterales is currently one of the most relevant problems in human medicine.

Several studies have evaluated the intestinal carriage of these bacteria in companion dogs, but their occurrence in AAI-animals has been poorly studied. Both companion- and AAI-dogs can be reservoirs of these resistant bacteria and, in some cases, the sharing of epidemiologically related strains between humans and dogs has been shown. It is more difficult to establish the direction of the transmission. Furthermore, transmission can occur directly between humans and animals, but it is also possible that both acquire the pathogen from a common environmental or food source.

The animal-assisted interventions produce a wide range of beneficial effects in patients, so the number of healthcare facilities that implement these interventions is constantly increasing. Thus far, no studies have confirmed the acquisition of infections during AAI sessions. The correct application of standardized protocols for infection control and prevention can allow the benefits of these interventions outweigh the risks.

Future research directions may concern longitudinal studies with sequential sampling from dogs, owners, and other family members and from the environment and food, in order to infer the dynamics of transmission.

## Data Availability

Data are contained within the text.
